# Are Cyanotoxins the Only Toxic Compound Potentially Present in Microalgae Supplements? Results from a Study of Ecological and Non-Ecological Products

**DOI:** 10.3390/toxins12090552

**Published:** 2020-08-28

**Authors:** Elisabet Sánchez-Parra, Soumia Boutarfa, Marina Aboal

**Affiliations:** 1 Laboratory of Algology, Faculty of Biology, Espinardo Campus, Murcia University, E-30100 Murcia, Spain; elisabet.sanchez@um.es (E.S.-P.); boutarfa_soumia@yahoo.fr (S.B.); 2 Faculty of Science of Nature and Life, University Abbes Laghrour, Khenchela 4004, Algeria

**Keywords:** anatoxins, bacteria, cyanotoxins, fungi, heavy metals, microalgae supplements, microcystins

## Abstract

Food supplements with microalgae are becoming increasingly abundant and can be easily found anywhere. The most popular products are based on cyanophytes, such as *Aphanizomenon flos-aquae*, *Arthrospira platensis* and *Limnospira maxima*, or on chlorophytes, such as *Chlorella* or *Haematoccus.* Although they are all advertised as being very beneficial for health, these products might be harmful because they may contain cyanotoxins and other contaminants, and no information on production methods or strain origins is usually provided. While legislation on the presence of microcystins in waters for different uses is clear, toxicological analyses are not compulsory for food supplements, nor for analyzing anatoxins. Given the potential risk of eating contaminated food, cyanotoxins, heavy metals and the presence of other contaminant organisms were analyzed in 10 microalgae food supplements. Microcystin-LR and anatoxin-a were detected in three analyzed products, and in both cyanophyte- and chlorophyte-based products. The light microscope study revealed the presence of different potentially harmful microbial contaminants. The ICP (OES) analyses detected high concentrations of some heavy metals, especially Pb. The results emphasize the need to promote the better control of food products containing microalgae, and to develop standard methodologies to analyze cyanotoxins and potential toxic compounds to protect consumer health.

## 1. Introduction

The increasing popularity of microalgae food products and supplements worldwide is based on their potential health benefits because they can represent a source of proteins, vitamins, essential fatty acids and antioxidants [1]. Current food production challenges, especially meat, and their consequences for our planet may explain why many people are changing to vegan or vegetarian diets [2]. Thus, meat substitutes like microalgae flourish [3,4], and the world demand for these products increases [1], even if the long-term success of such products depends especially on their digestibility, bioavailability and clinical evidence [5].

Cyanobacteria are considered natural health promoters in different ways: prevention and control of obesity, control of diabetes, as anti-inflammatory, antibacterial, antiviral, anticancer, hypocholesteraemic or hypotrigliceridaemic agents, as antioxidants, and as promoters of the immune system and neuroprotectors, as several clinical trials have shown [6,7,8,9,10,11,12,13,14,15,16]. BGAS (Blue Green Algal Supplements) can be easily obtained in herbalists, supermarkets, pharmacies or on the Internet [6]. The most frequent species used for these food supplements are *Limnospira maxima* (Setchell and Gardner) Nowicka-Krawcysk, Mühlsteinova and Hauer (usually under the name of *Spirulina maxima*), *Arthrospira platensis* Gomont (normally called *Spirulina platensis* (Gomont) Geitler) and *Aphanizomenon flos-aquae* Ralfs ex Bornet and Flahault [17,18]. Microalgae from other taxonomic groups are also frequently consumed, of which the most popular are probably *Chlorella* and *Haematococcus* (Trebouxiophyceae and Chlorophyceae, respectively). These products are available in diverse forms (e.g., pills, capsules, powder, flakes, tablets) [19,20,21] and have been progressively included in other products, like biscuits, pasta, drinks or yoghurt [22,23]. As it is compulsory to only indicate the composition, daily intake and a warning about them not substituting healthy diet in most countries [24,25], the labels of these products are often inaccurate and do not offer information about geographic origin, if they are collected from nature, or if they are produced in close bioreactors or open systems. All these aspects represent a potential hazard and a risk for different contamination kinds.

When microalgae are produced in outdoor ponds, they can be affected by air-coming pollutants, and the presence of other algae and microorganisms is unavoidable [26,27] and poses a hazard if these organisms produce toxic compounds. The presence of *Microcystis aeruginosa* (Kützing) Kützing (the most important microcystin producer) in supplements of *Aphanizomenon flos-aquae* Ralfs ex Bornet and Flahault has been documented [28,29], but sometimes those containing *A. flos-aquae* are used to treat children with hyperactivity or attention deficiency syndrome [30]. Thus no clear idea is available of what a healthy daily dose actually is [17,21,30,31]. These products are normally bought with no medical prescription and are perceived as being safe [6,25,32,33]. However, some studies have detected microcystin (MC) concentrations below 1 µg/g [6,30,33,34,35], and chronic exposure to this low concentration can also pose a major health problem, especially for children given its carcinogenic character.

The WHO recommends a tolerable daily intake (TDI) of 0.04 µg/kg for adults of corporal body/day microcystin, which means 0.24 µg per day for a person weighing 60 kg [36]. In some countries, however, this limit has been modified, e.g., the USA (Oregon), 1 µg of MC-LR equivalents/g of dry weight has been proposed [37]. There is also growing concern about high levels of some metals, mostly related to the location of the cultivation ponds or chemicals used for harvesting biomasses [38], or even essential macro- and trace elements that can pose health problems [39]. The presence and proliferation of other microorganisms (fungi and bacteria) are frequently detected in food [40], especially under deficient conservation conditions. Food supplements are not free of such contamination and no data on it are available.

This study aims to assess the ecological and non ecological microalgae product contents in cyanotoxins, other microbial organisms, toxic metals and macro- and trace elements to envisage the importance of control during production processes and labeling, and the need for international legislation.

## 2. Results

### 2.1. Microscopic Analysis

**Figure 1:**
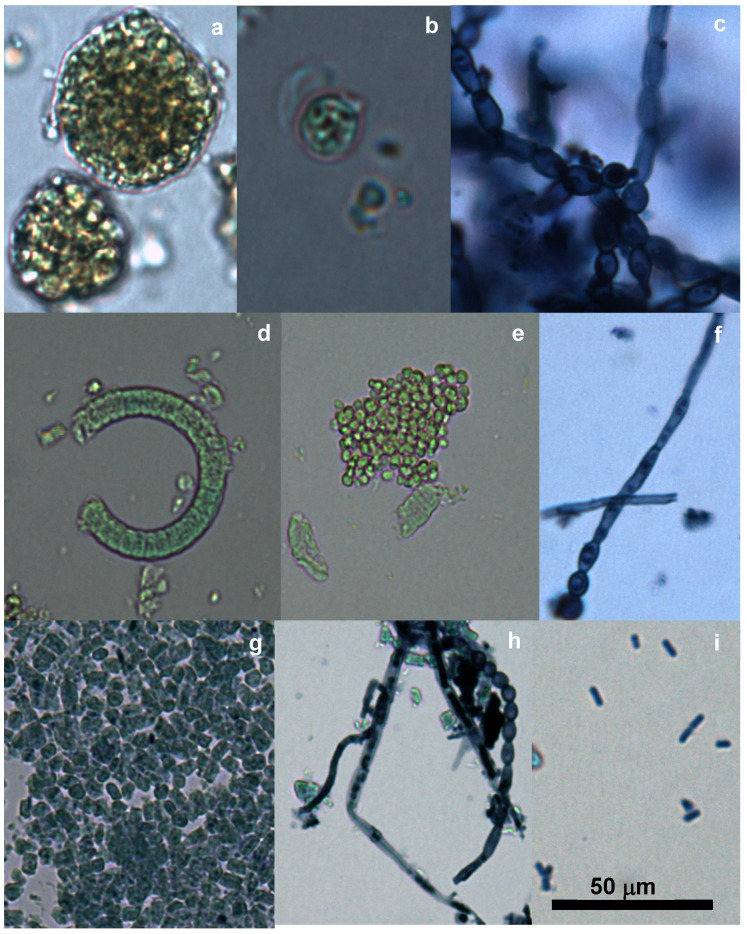
Microscopic observation of the microalgae supplement products: (**a**). Cell aggregations (product 7); (**b**) *Trebouxia*-like cells (product 7); (**c**) Branched fungal hyphae (product 7); (**d**). *Arthrospira* fragment (product 5); (**e**). *Chlorococaceae* and *Arthrospira* fragments (product 8); (**f**). Fungi hypha and conidia (product 3); (**g**) *Aphanizomenon* phragmented (product 10); (**h**). Fungi hypha and conidia (product 9); (**i**) *Synechococcus* (product 10). The material from image 1 was stained with lugol and that from images 3, 6, 8 and 9 was stained with methylene blue.

**Figure 2:**
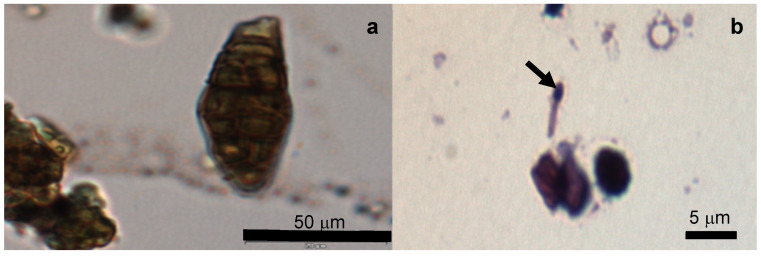
Microorganisms observed in the analyzed microalgae supplement product 7: (**a**) Multicellular fungi conidium; (**b**) *Clostridium* type Gram- endospore (arrow).

All the samples contained lower or higher proportions of microorganisms other than microalgae, of which fungi hyphae, fungi conidia, other algae and bacteria were frequent (Figure 1 and Figure 2). Chlorophyta were present in the *Spirulina* samples, and *Spirulina*-like morphos were also found in the *Chlorella* products. *Chlorella* product number 7 contained different chlorophytes (*Trebouxia*-like) and several cyanophytes, diatoms, fungi hyphae and conidia. Gram-staining allowed the *Clostridium* with endospores in this product to be identified (Figure 2). The proportion of these contaminants varied from low to high (Table 1). The variability between the studied aliquots was always very wide. The ecological products contained a higher proportion of contaminants and were the only ones with *Clostridium*.

### 2.2. ELISA

Of the samples, 30% were positive to microcystin/nodularin, namely products 7 (Chlorella), 9 (Klamath and Spirulina) and 10 (Upper Klamath Algae), and some had higher concentrations than the standard range (Table 2).

### 2.3. HPLC-MS

The three samples positive with ELISA contained MC-LR at different concentrations. However, in product 7 (*Chlorella*), the concentration was unquantifiable as it was under the limit of quantification (LoQ; Table 3), but its presence was confirmed in the fortified samples. Anatoxin-a was also present in the three samples. These, and all the other tested variants ([Dhb^7^]-MC-LR, MC-RR, MC-YR, MC-LW and MC-LF) and nodularin, were below the detection level in the other analyzed products.

**Figure 3:**
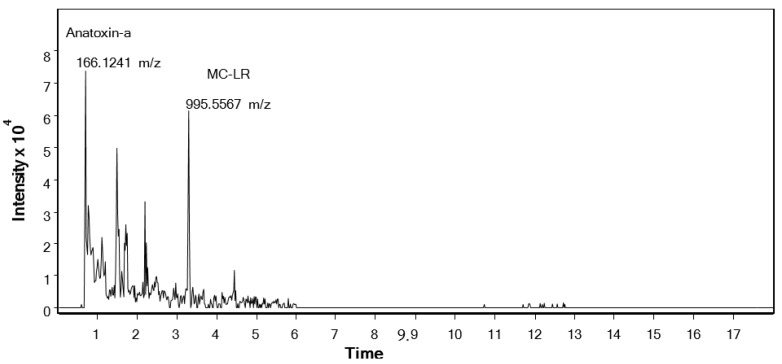
Chromatogram of *A. flos-aquae* and *S. platensis* (product 9).

The chromatograms of product 9, containing *Aphanizomenon* and *Spirulina*, with labeled Anatoxin-a (ANA-a) and Microcystin LR (MC-LR) peaks, are shown in Figure 3.

### 2.4. Elemental Composition

The concentration of metals vastly varied among products (Table 4). The Al and Fe concentrations were very high in products 1, 3 and 6, and Cr was high in products 1 and 3. Cu was high in 7 compared to all the other products, but its levels fell within the safety limits according to the European Food Safety Authority (EFSA) [41]. Pb was detected in products 1–3, 5–6 and 8. As was found in products 6, 9 and 10, Mo only in samples 6 and 10, Ti was very high in 1, 3 and 6, and Ni was high only in sample 3 compared to the rest. In all cases, levels fell within the safety limits [41].

The Na concentration was very high in samples 1 and 3 versus all the others, which probably indicates a marine culture (Table 4). However, if the products’ intake recommendation is followed (Table 5), doses do not reach the maximum ones recommended by European regulations [41]. The recommended dietary daily intake for an adult male is estimated at between 8–10 mg Fe/day [42,43]. Products 1–3 and 6 exceeded that value, especially product 1 which, according to the indications on its label, it could represent 5× the recommended value.

The full elemental analysis results are shown in the Supplementary Material (Table S1).

## 3. Discussion

As it is not compulsory, most commercial products do not display information about the origin of their material, culture, collection or preservation methods. Only product number 2 indicated that the alga was grown in freshwater ponds, and products 9 and 10 explain that *Aphanizomenon* was collected from Klamath Lake, but they added *Spirulina* of unknown origin to product 9. On other occasions, statements were untrue because, as in products 1 and 2, they indicated that they were free of heavy metals or bacteria. However, fairly high concentrations of Pb and bacteria were detected in the former. No information about the presence of other organisms was displayed because it is not compulsory to do so. Our microscopic study showed the presence of several different microorganism species in all the products, especially in 7 (*Chlorella*), including some potential cyanobacteria. The presence of foreign species (not indicated on labels) was more frequent in the ecological than in the non-ecological products.

The presence of fungi hyphae and conidia was frequent, and a good number of fungi could produce toxic compounds, and can even prove lethal [41]. Endospores of an unidentified *Clostridium* were also detected, and some species of this genus could also produce lethal compounds [44]. The proliferation on these organisms is probably related to inadequate microalgae biomass preservation after collection [40].

The presence of microcystin in microalgae-based food supplements has been previously documented, especially when they contain *Aphanizomenon* [6,21,27,28,33,35,45,46]. The more commercialized *Aphanizomenon* comes from Klamath Lake (USA), and several reports about the toxicity of this product are found in literature [28,29,30,46]. Anatoxin-a has been also detected in food supplements previously [47,48]. When microalgae production takes place outdoors, it is almost impossible to avoid the presence of other species, and some can produce toxins regardless of the growing algae being cyanophytes or not. According to our results, both MC-LR and anatoxin-a were detected in a *Chlorella* product, as formally indicated in [30] and [49].

The provided labels are usually inaccurate as factual and declared contents usually do not match, although other authors have found a stronger resemblance [38,50]. The public is not generally aware that ecological labels are not a non-toxicity guarantee because they only indicate that chemical fertilizers and pesticides were avoided during production. The analyzed subsamples offered wide variability, which indicates that both toxin concentration and the concentration of contaminants can change with different doses.

The microcystin TDI for adults must be below 0.04 µg per kg of body weight, which means a maximum intake of 2.4 µg for people weighing 60 kg [51]. If product recommendations are followed, the limit will not be surpassed in this case. However, the risk remains, especially for children if we bear in mind these compounds’ carcinogenic characters and their longer exposure to them. No recommendations exist for anatoxin-a.

The WHO [51] does not recommend eating food containing Pb, which emphasizes that care should be taken with children because it is apparently related to neurodegenerative processes, Moreover, Pb is absorbed more in children than in adults, and accumulates in soft tissues and bones. Nevertheless, a high concentration of this element has already been reported [38]. The EFSA [52] recommends a dose of 0.50 mg/kg body weight for children up to the age of 7 years, but information about the presence of this metal is usually omitted from labels. Sr and Ti are used as food conservative products and are not considered problematic. The same applies to Al, which is employed as a flocculant [53], but the EFSA recommends further studies being conducted on the potential risk of being exposed to the increasing Al levels detected in food [52]. Some products have a high Fe concentration and poisoning with oral pharmaceutical-like iron preparations can cause mucosal erosion in both the stomach and intestine, with young children particularly at risk [42,43]. Given the potential pro-oxidant effects of Fe, extensive research into possible links between Fe and cancer development has been carried out. Several case-control studies show that the risk of colorectal cancer is positively associated with Fe intake [43,54,55].

In some cases, a product’s chemical composition is displayed. However, major elements like Na^+^ are not included, not even when some strains are grown in saline water that increases the probability of a high concentration of this ion, which poses a risk for people with heart or hypertension problems, especially when products with this microalga are considered hypotensive [9].

Overall the most important problem is that daily doses for children are not usually indicated, which increases this population’s vulnerability, especially when these products have been recommended for different child illnesses.

## 4. Conclusions

Microalgae products may contain microcystins that increase the probability of developing cancer, especially as microcystins are carcinogenic and these products are consumed on a daily basis. The TDI for adults and children should be clearly indicated on labels and take into account children’s potentially longer exposure, especially when these products are recommended for several child illnesses.

Anatoxin-a has also been detected, which confirms previous reports urging legislative regulations being made for this cyanotoxin.

Some products contain other biological contaminants like fungi and bacteria, some of which are toxic and increase the probability of toxic events.

The presence of heavy metals and other potentially harmful ions was detected in most products, which poses increasing toxicity risks for consumers.

An international agreement about providing a toxicological analysis of products should be compulsory, whose results ought to be indicated on labels, together with information about production and preservation methods and/or elemental analyses.

## 5. Materials and Methods

### 5.1. The Analyzed Algae Supplement Products

Products were selected from those containing cyanobacteria and *Chlorella* by ecological (5) and non-ecological production (5) (Table 6). All the products were bought in local shops, except for the *Aphanizomenon*-containing products, which were purchased on by the Internet.

### 5.2. Light Microscopy

All the samples were observed before any later analysis was done under an OLYMPUS BX50F microscope equipped with a digital camera. Samples (500 mg) were suspended in deionized water and several stain methods were followed: methylene blue to evidence sheaths or mucilage; lugol (IIK) to differentiate starch/pyrenoids; Gram to observe bacterial walls. Three slides were prepared per sample and the microorganisms present in three random traverses from each slide were quantified on a semiquantitative scale from 1 to 3: 1= 1–20 %, 2= 21–50%, 3>50%.

### 5.3. Extraction

Samples were ground in a mortar with a Teflon tissue grinder and were weighed on a precision balance. The recorded weights are found in Table 2 and Table 3. Three replicates were prepared per sample.

Protocols [56] and [57] were followed for extraction purpose. Samples were transferred to glass tubes and left in a freezer at −20 °C for 1 h before being transferred to a freezer drier (Christ Alpha 1-2 LD) for 2 h. The extraction process was repeated three times at 60 °C in 2.5 mL 75% methanol-25% Millipore water (*v*/*v*). Extracts were dried in a Speedvac (Savant SPD 121P, Waltham, MA, USA), reconstituted in 900 mL methanol, transferred to 2 mL Eppendorf vials with a cellulose-acetate filter (Corning Costar Spin-X centrifuge tube filters) and centrifuged for 5 min at 16,000× *g* (Optima L-100 XP de Beckman Coulter, Brea, CA, USA). Filtrates were transferred to amber glass vials for the HPLC/MS analysis.

### 5.4. Cyanotoxins Characterization and Quantification

#### 5.4.1. ELISA Test

Extracts were analyzed following the recommendations of Abraxis (Los Ángeles, CA, USA) Microcystins (ADDA-DM ELISA kit PN522015 Microtiter Plate). The results were obtained after reading with a plate reader BMG Labtech FLUOstar Omega at 450 nm. The detection limit of the kit was 0.10 µg/L for MC-LR.

#### 5.4.2. HPLC-MS

All the extracts were later analyzed by HPLC Agilent 1290 Infinity II coupled to a hybrid mass spectrophotometer Agilent Q-TOF 6550, with an ionization source JetStream electrospray + i-Funnel. Samples were analyzed for eight MC variants ([Dhb^7^]-MC-LR, MC-RR, MC-YR, MC-LR, MC-LW, MC-LF, anatoxin-a (ANA) and nodularin (NOD) from SIGMA-ALDRICH). Compounds were separated in an Agilent Eclipse XDB-C18 4.6 × 150 mm, 5 mm column by Millipore water with 0.1 % formic acid (*v*/*v*, eluent A) and acetonitrile with 0.1% formic acid (*v*/*v*, eluent B). The elution program was 0–2 min 30% B, 6–12 min 90% B, with a linear increase of B between 2 and 6 min, and a 5-min post run at 30% B. The injection volume, flow and column temperature were 20 mL, 0.5 mL/min and 40 °C, respectively. MS operated in the positive mode and nitrogen was used as the drying and collision gas. The quadrupole was operated in the unit mode and four spectra/sec were recorded. Samples were quantified against a calibration curve and subsequently corrected for recovery (Table 7). Two replicas were obtained per sample and each replica was injected once.

The presence of phenylalanine was discarded because its theoretical m/z was 166.0863 and the employed method could separate it easily from anatoxin [58].

### 5.5. Elemental Composition (ICP-OES)

The ground samples (500 mg) were digested in an ultraclave microwave digestor (Milestone Inc. Shelton, USA) and then analyzed with an ICP-OES ICAP 6500 DUO Thermo in the CEBAS-CSIC Ionomic Laboratory. The detection level was 0.01 µg/g.

## Figures and Tables

**Table 1:** Proportion of the microorganisms observed in the analyzed microalgae supplement products: 1 = 1–20%, 2 = 21–50%, 3>50%, - absent.

	Products Commercial Name									
Contaminants	1 Spirulina	2 Chlorella	3 Alga Spirulina	4 Espirulina	5 Spirulina	6 Spirulina	7 Chlorella	8 Spirulina	9 Klamath and Spirulina	10 Upper Klamath Algae
Fungi hyphae	3	3	3	2	2	2	2	2	3	3
Conidia	2	2	2	1	1	2	2	1	1	1
Other algae	1	1	1	1	1	1	2	1	1	1
Bacteria	2	2	2	2	2	2	2	2	2	2
Bacteria endospores	-	-	-	-	-	-	1	-	-	-

**Table 2:** ELISA test results: <0.15 below the value of the lowest concentrated standard, >5.00 = over the value of the highest concentrated standard.

	Products Commercial Name									
Toxins	1 Spirulina	2 Chlorella	3 Alga Spirulina	4 Espirulina	5 Spirulina	6 Spirulina	7 Chlorella	8 Spirulina	9 Klamath and Spirulina	10 Upper Klamath Algae
Absorbance 450 nm	0.761 ± 0.051	1.042 ± 0.126	0.759 ± 0.087	0.868 ± 0.071	0.867 ± 0.048	0.719 ± 0.107	0.451 ± 0.132	0.960 ± 0.180	0.115 ± 0.004	0.172 ± 0.012
MC-NOD (μg)	<0.15	<0.15	<0.15	<0.15	<0.15	<0.15	0.813	<0.15	>5.00	>5.00
Dry weight (g)	0.5	0.5	0.5	0.5	0.5	0.5	0.5	0.5	0.5	0.5

**Table 3:** HPLC-MS analysis results. Limit of quantification (LoQ).

Product	MC-LR (µg/g)	ANA-a (µg/g)	Dry Weight (g)
7. Chlorella	<LoQ	0.034 ± 0.002	1.64
9. Klamath and Spirulina	0.008 ± 0.002	0.025 ± 0.006	1.57
10. Upper Klamath Algae	0.002 ± 0.0001	0.002 ± 0.001	1.53

**Table 4:** Elemental composition of microalgae products (detection level = 0.01 µg/g).

Products	Al µg/g	As µg/g	Cr µg/g	Cu µg/g	Fe µg/g	Mo µg/g	Na µg/g	Ni µg/g	Pb µg/g	Sr µg/g	Ti µg/g
1. Spirulina	308.65 ± 15.43	<0.01	2.03 ± 0.06	1.22 ± 0.09	940.55 ± 47.02	<0.01	12.55 ± 0.75	0.70 ± 0.04	0.54 ± 0.04	23.06 ± 2.07	22.93 ± 0.91
2. Chlorella	43.62 ± 1.31	0.13 ± 0.05	1.19 ± 0.06	3.52 ± 0.14	661.64 ± 46.31	<0.01	0.41 ± 0.02	0.38 ± 0.03	0.26 ± 0.01	7.26 ± 1.09	9.99 ± 0.79
3. Alga Spirulina	482.53 ± 19.30	<0.01	2.74 ± 0.14	1.63 ± 0.03	1053.56 ± 84.28	<0.01	8.24 ± 0.41	2.54 ± 0.20	0.87 ± 0.03	24.37 ± 1.70	35.84 ± 1.79
4. Espirulina	39.08 ± 0.78	<0.01	0.62 ± 0.03	0.30 ± 0.02	324.34 ± 9.72	<0.01	1.66 ± 0.03	0.08 ± 0.01	<0.01	15.24 ± 0.45	2.42 ± 0.12
5. Spirulina	40.76 ± 1.63	<0.01	0.41 ± 0.02	0.45 ± 0.04	260.45 ± 15,63	<0.01	11.19 ± 0.78	0.20 ± 0.01	0.14 ± 0.01	12.47 ± 0.75	1.96 ± 0.05
6. Spirulina	263.73 ± 18.48	0.34 ± 0.02	1.83 ± 0.11	0.99 ± 0.06	896.61 ± 44.83	3.15 ± 0.19	2.26 ± 0.14	0.26 ± 0.02	0.39 ± 0.03	13.27 ± 0.39	34.81 ± 2.44
7. Chlorella	17.58 ± 0,88	<0.01	1.06 ± 0.04	9.14 ± 0.73	123.59 ± 2.47	<0.01	2.15 ± 0.13	0.16 ± 0.01	<0.01	20.36 ± 1.42	1.66 ± 0.06
8. Spirulina	77.40 ± 5.42	<0.01	1.11 ± 0.08	0.65 ± 0.04	262.05 ± 20.96	<0.01	8.14 ± 0.73	0.54 ± 0.02	0.14 ± 0.01	27.56 ± 1.10	3.72 ± 0.33
9. Klamath and Spirulina	97.57 ± 6.83	2.07 ± 0.10	0.79 ± 0.04	2.85 ± 0.19	363.18 ± 21.79	0.36 ± 0.03	3.48 ± 0.28	0.66 ± 0.02	<0.01	25.42 ± 1.52	6.14 ± 0,25
10. Upper Klamath Algae	20.01 ± 1.80	2.20 ± 0.06	0.28 ± 0.02	3.68 ± 0.15	305.89 ± 12.24	2.03 ± 0.08	1.82 ± 0.11	0.32 ± 0.01	<0.01	33.47 ± 2.67	2.82 ± 0.14

**Table 5:** Recommended daily intake (RDI) from product labels and the corresponding daily intake for a person weighing 60 kg (n.d. below the detection level= 0.01 μg/g).

Products	RDI G	Al mg	As µg	Cr µg	Cu µg	Fe mg	Mo µg	Na µg	Ni µg	Pb µg	Sr mg	Ti mg
1. Spirulina	60.00	18.51	n.d.	121.80	73.20	56.43	n.d.	753.00	42.00	32.40	1.38	1.37
2. Chlorella	25.00	1.10	3.25	29.75	88.25	16.54	n.d.	10.25	9.50	6.50	0.18	0.25
3. Alga Spirulina	10.00	4.82	n.d.	27.40	16.30	10.53	n.d.	82.40	25.40	8.70	0.24	0.36
4. Espirulina	0.90	0.04	n.d.	0.56	0.27	0.29	n.d.	1.49	0.07	n.d.	0.01	0.002
5. Spirulina	2.34	0.09	n.d.	0.96	1.05	0.61	n.d.	26.18	0.47	0.33	0.03	0.004
6. Spirulina	3.60	0.95	1.22	6.59	3.56	3.22	11.34	8.14	0.94	1.40	0.05	0.12
7. Chlorella	3.60	0.06	n.d.	3.82	32.90	0.44	n.d.	7.74	0.58	n.d.	0.07	0.005
8. Spirulina	1.35	0.10	n.d.	1.50	0.88	0.35	n.d.	10.99	0.73	0.19	0.04	0.005
9. Klamath and Spirulina	1.20	0.12	2.48	0.95	3.42	0.43	0.43	1.50	0.79	n.d.	0.03	0.007
10. Upper Klamath Algae	0.75	0.01	1.65	0.21	2.76	0.23	1.52	1.37	0.24	n.d.	0.02	0.002

**Table 6:** Displayed composition, format, cultivation and country of origin of the analyzed products.

Product Number	Product Name	Microalgae Composition	Producer/Seller	Ecological	Presentation	Cultivation	Country of Origin
1	Spirulina	100% *Spirulina*	Ecolife	Yes	Powder	Outdoor ponds	China
2	Chlorella	100% *Chlorella*	Ecolife	Yes	Powder	Outdoor ponds	China
3	Alga Spirulina	*Arthrospira platensis*	Drasanvi	Yes	Powder	-	Spain
4	Espirulina	71% *Limnospira maxima*	Vive+ Salud y Vida	No	Capsules	-	Spain
5	Spirulina	*Arthrospira platensis*	Biogran S.L.	No	Tablets	-	-
6	Spirulina	100% *Spirulina*	Raab Vitalfood	Yes	Tablets	Controlled biologic aquaculture	No EU
7	Chlorella	100% *Chlorella*	Raab Vitalfood	Yes	Tablets	Controlled biologic aquaculture	No EU
8	Spirulina	*Arthrospira platensis*	Nature Essential	No	Tablets	-	No EU
9	Klamath and Spirulina	50% *Aphanizomenon flos-aquae* 50% *Arthrospira platensis*	Santiveri	No	Tables	Collected in nature + ?	-
10	Upper Klamath Algae	*Aphanizomenon flos-aquae*	Blue Green Planet	No	Capsules	Collected in nature	USA

**Table 7:** Technical characteristics of the HPLC-MS analysis.

Toxin	Formula	Theoretical *m/z*	Standard *m/z*	Standard Rt(min)	Experimental *m/z*	Experimental Rt(min)
Anatoxin-a	C_10_H_15_NO	166.1226	166.1221	0.52	166.1219	0.58
Nodularin	C_41_H_60_N_8_O_10_	825.4505	825.451	2.39	-	-
[Dhb^7^]-MC-LR	C_48_H_72_N_10_O_12_	981.5404	981.5404	3.39	-	-
MC-LR	C_49_H_74_N_10_O_12_	995.556	995.5567	3.31	995.5528	3.3
MC-LW	C_54_H_72_N_8_O_12_	1025.5342	1025.5342	4.71	-	-
MC-RR	C_49_H_75_N_13_O_12_	1038.5731	1038.5731	4.70	-	-
MC-YR	C_52_H_72_N_10_O_13_	1045.5353	1045.5353	3.16	-	-

## Supplementary Materials

The following are available online at https://www.mdpi.com/2072-6651/12/9/552/s1, Table S1: Complete elemental analysis of microalgae supplements analysed with ICP/OES (Detection level 0.01 μg/g).
